# Supplementary material for: Asynchronous magnetic resonance elastography: Shear wave speed reconstruction using noise correlation of incoherent waves
Source: Magn Reson Med. 2022 Oct 27;89(3):990–1001. doi: 10.1002/mrm.29502 (PMC9792433; doi:10.1002/mrm.29502)
Supplement: Supplementary file 1 — DATA S1 MATLAB code used to generate the numerical simulations of Figure 1 [file MRM-89-990-s002.zip › k-Wave/helpfiles/example_tvsp_angular_spectrum.html]

Holographic Projections Using The Angular Spectrum Method Example (k-Wave)


# Holographic Projections Using The Angular Spectrum Method Example

This example demonstrates how to project time-domain data between parallel planes using the angular spectrum method. It builds on the Simulations In Three Dimensions Example.

- Open the file in the MATLAB Editor
- Run the file in MATLAB

## Contents

- Overview of the angular spectrum method
- Generating the input and output data
- Acoustic holography using the angular spectrum method

## Overview of the angular spectrum method

Given time-series data measured over a plane, it is possible to project this data to other parallel planes using the angular spectrum method (sometimes called the angular spectrum approach). This method works by first decomposing the time signals into a series of frequency components (with a given amplitude and phase) by taking a 1D Fourier transform in time. For each frequency component, the amplitude and phase across the plane is then decomposed into a series of planes waves by taking a 2D Fourier transform in space. Each plane wave component is then propagated to the next plane by multiplying by a spectral propagator. The complex pressure at the next plane is reconstructed by taking an inverse Fourier transform in space. After propagating all temporal frequency components, the time-series at the next plane is then reconstructed.

If the time series data is known over an infinite plane, in principle, the angular spectrum method can be used to project this data to any other parallel plane without any loss in accuracy (excluding the contributions from evanescent waves). However, in practice usually the time series data is only measured over a finite plane that captures most of the wave energy. This is the case when experimentally measuring the output from a transducer using a hydrophone. In this example, the time series data over two parallel planes is simulated using `kspaceFirstOrder3D`. The function `angularSpectrum` is then used to project the data from the first plane to the second, and the projected data is compared with the simulated data. Note, for a single frequency, the function `angularSpectrumCW` could instead be used.

## Generating the input and output data

The function `kspaceFirstOrder3D` is used to simulate the output from a focused bowl transducer created using `makeBowl`. The transducer is oriented in the z-direction, and the sensor mask is defined as two parallel x-y planes. To allow easy quantitative comparison with `angularSpectrum` (which computes the projected time series in retarded time), the time step is adjusted so the distance between the two planes is an integer number of time steps. A snapshot of the simulation is shown below.


## Acoustic holography using the angular spectrum method

The function `angularSpectrum` is used to project the input data from the first plane to the second plane in a single step. To improve the accuracy of the projection, the grid is expanded using the optional input `'GridExpansion'` (the same behaviour can also be obtained by directly setting the FFT size using the optional input `'FFTLength'`). This increases the number of discrete spatial frequencies used in the projection.

```
% run projection from the first plane to the second plane using the angular spectrum method
[~, plane_2_as] = angularSpectrum(plane_1_kw, dx, kgrid.dt, proj_dist, c0, 'GridExpansion', 50);
```

The projected data is then compared with the data simulated using `kspaceFirstOrder3D`. The plots below show different visualisations of the time domain waveforms, including the raw time series data, a maximum intensity projection through the time axis, and the time signal at the centre of the plane. There is close agreement between to the two datasets.

Without the grid expansion, the maximum difference increases from ~1% to ~3%. However, further increasing the size of the grid expansion has diminishing returns, and will not reduce the difference to zero. This is because the plane of measured data does not capture the entire wave-field. To further reduce the difference, the size of the measured plane must be increased.
